# Longitudinal multimodal imaging in mild to moderate Alzheimer disease: a pilot study with memantine

**DOI:** 10.1136/jnnp.2007.141648

**Published:** 2008-06-27

**Authors:** R Schmidt, S Ropele, B Pendl, P Ofner, C Enzinger, H Schmidt, A Berghold, M Windisch, H Kolassa, F Fazekas

**Affiliations:** 1 Department of Neurology, Medical University of Graz, Austria; 2 Divisions of Neuroradiology and Nuclear Medicine, Medical University of Graz, Austria; 3 Department of Radiology, Institute for Medical Informatics, Statistics and Documentation, Medical University of Graz, Austria; 4 Institute for Molecular Biology and Biochemistry, Medical University of Graz, Austria; 5 JSW Research, Forschungslabor GmbH, Graz, Austria; 6 Merz Pharma Austria GmbH, Vienna, Austria

## Abstract

**Objective:**

To study the feasibility of multimodal neuroimaging in mild to moderate Alzheimer disease (AD) and to estimate the size of possible treatment effects of memantine on potential functional, structural and metabolic biomarkers of disease progression.

**Methods:**

In this randomised, double-blind, placebo-controlled pilot study, 36 patients with moderate AD received 52 weeks of memantine (20 mg/day) or placebo. Patients were re-evaluated after 26 and 52 weeks to measure the change from baseline in several outcome measures including global and regional glucose metabolism, total brain and hippocampal volumes, as well as chemical shift imaging-derived global and regional *N*-acetylaspartate and myoinositol concentrations.

**Results:**

In the total population, global glucose metabolism decreased by 2.3% (p<0.01), total brain volume by 2.1% (p<0.001) and hippocampal volume by 2.7% (p<0.01) after 52 weeks. Chemical shift imaging (CSI) spectra were severely affected by patient-induced artefacts and highly variable. Patients receiving memantine showed less decline in glucose metabolism in all brain areas than patients on placebo. Their loss of hippocampal volume was substantially smaller (2.4% vs 4.0%). No between-group differences were seen for changes in total brain volume.

**Conclusions:**

The results support the use of multimodal imaging including MRI and positron emission tomography (PET) to monitor the progression of moderate AD. CSI yielded unreliable longitudinal results. The data suggest that memantine has potentially protective effects in AD and they can be used for planning larger confirmatory studies on the cerebral effects of memantine.

Current treatments in Alzheimer disease (AD) apparently do not slow the disease.^1^ Therapies that modify AD by interfering with the underlying neurodegeneration are under investigation.^2^ Neuroimaging markers that substantiate disease-modifying effects are attractive investigational targets.^3^ ^4^ The rate of whole brain and hippocampal volume loss, longitudinal changes in *N*-acetylaspartate (NAA), choline and myoinositol (MI) concentrations, and decline in brain perfusion and metabolism, are potential imaging endpoints for therapeutic trials.^4^ They reportedly correlate with the severity of histopathology^5^^–^8 and cognitive performance.^4^

Few therapeutic trials have implemented these measures in patients with AD and most of them used a single modality approach.^9^^–^14 This provides a restricted view on disease-related changes over time and considers only selected aspects of treatment effects although these may be manifold, including brain metabolism, function and structure. These different aspects may now be appreciated by specific imaging technologies, but the feasibility and contribution of long-term multimodal imaging to study therapeutic responses in AD has not yet been sufficiently explored. Knowledge of long-term change in different imaging measures and assessment of the variability of results in patients with AD are a prerequisite for the use of such methods in treatment trials, as is reproducibility assessment. Such data could be obtained from a purely observational study unclouded by possible therapeutic effects. However, it is difficult to conduct long-term studies in patients with AD without offering them any treatment. We therefore performed a 1-year pilot feasibility study on multimodal imaging in mild to moderate AD coupled with specific treatment. We determined the longitudinal changes and their variability on ^18^F-fluorodeoxyglucose (FDG) positron emission tomography (PET), chemical shift imaging (CSI) and 3D MRI in patients with AD who had been randomised to receive either memantine or placebo.

## PATIENTS AND METHODS

### Patients

Patients over 50 years old were eligible if they had a diagnosis of probable AD according to the Diagnostic and Statistical Manual of Mental Disorders (DSM)-IV^15^ and National Institute of Neurological and Communicative Disorders and Stroke and the Alzheimer's Disease and Related Disorders Association (NINCDS–ADRDA) criteria,^16^ a Hachinski score ⩽4,^17^ and an Mini-Mental State Examination (MMSE) score between 14 and 22.^18^ When we started the study, cholinesterase inhibitors were approved in Austria for mild to moderate AD (MMSE 12–24), and memantine for moderately severe and severe AD (MMSE 3–14). We considered a placebo group to be crucial but did not want to exclude study participants from approved treatments. Therefore, we included only those patients who (1) had either failed to respond to cholinesterase inhibitors or experienced severe side effects leading to termination of such treatment and (2) had MMSE scores >14, which, at the time of study conduct, had excluded them from other approved antidementia treatment once cholinesterase inhibitors had been stopped. To avoid withholding licensed therapy from study participants, we a priori defined that, whenever a participant worsened clinically obtaining an MMSE score <15, he/she would be switched to active treatment without breaking the double-blind code and remain in the study. This applied to three cases in the placebo group.

None of the patients included could obtain licensed treatment at study entry or would be withheld such treatment during the study, which prompted the local ethics committee to approve a 1-year placebo-controlled trial. Cholinesterase inhibitor treatment had to be terminated at least 4 weeks before screening. In addition, patients had to be in generally good health, ambulatory and with sufficient hearing and vision for compliance with testing procedures. Only patients able to undergo MRI were enrolled. Patients with a primary diagnosis of psychiatric disorders other than AD, cerebrovascular disease, or any unstable medical condition were excluded. Patients were permitted to continue on stable doses of concomitant medications received at least 3 months before screening. These included low-dose atypical neuroleptics, selective serotonin re-uptake inhibitors, non-centrally active antihypertensives, anti-inflammatory drugs, platelet antiaggregants and anticoagulants, laxatives, diuretics and sedatives/hypnotics. Anticonvulsants, anti-Parkinson agents, barbiturates, Gingko biloba and nootropics, systemic corticosteroids and insulin were not permitted.

The study was carried out according to the Declaration of Helsinki. Written informed consent was obtained from the patients and their caregivers.

### Protocol

This was a single-centre, 52-week, randomised, double-blind, placebo-controlled, parallel-group pilot study conducted at the Medical University of Graz, Graz, Austria, between March 2003 and August 2005. Patients were randomly assigned by a computerised randomisation schedule to either placebo or memantine. Randomisation used a permuted block design and considered the presence or absence of an apolipoprotein-E-ϵ4 allele as a stratification criterion, because of previous data indicating more rapid decline in apolipoprotein-E-ϵ4 carriers.^19^ Briefly, two randomisation lists were generated, one for all patients carrying at least one apolipoprotein-E-ϵ4 allele and one for all other apolipoprotein-E genotypes, so that carriers and non-carriers were equally distributed between the two treatment groups.

Patients assigned to memantine were titrated to a dose of 20 mg/day over a 4-week period. Daily dose consisted of two identical tablets so as not to reveal the titration scheme: two placebo tablets throughout the study for patients treated with placebo and two tablets containing either 5 mg or 10 mg memantine depending on the titration stage for patients treated with memantine. Evaluation of imaging data was performed blinded to the patients’ clinical results.

We screened patients by medical history, physical and neuropsychiatric examination, laboratory assessment including apolipoprotein-E genotyping, MMSE,^18^ Geriatric Depression Scale (GDS),^20^ and modified Hachinski scale.^17^ All subjects had an electrocardiogram (ECG) and a brain computed tomography (CT) or MRI scan not older than 1 year. Screening was performed within 31 days before patients began double-blind treatment and verification of AD diagnosis was based on screening results. At baseline, we repeated the physical examination including vital signs, MMSE and laboratory tests. In addition, participants underwent psychometric testing including Alzheimer Disease Assessment Scale – cognitive subscale (ADAS-Cog),^21^ Clinical Dementia Rating (CDR),^22^ and Alzheimer Disease Cooperative Study – activities of daily living (ADCS-ADL) inventory ,^23^ and MRI, quantitative ^1^H-CSI and ^18^F-FDG PET. The first dose of study medication was then administered. Follow-up visits were scheduled at the end of weeks 12, 26 and 52. At each follow-up visit, routine physical examination, laboratory tests, psychometric testing, medication compliance check and adverse events monitoring were performed.

MRI and ^1^H-CSI were repeated at weeks 26 and 52; PET scanning was repeated at the end of week 52, only. Patients who withdrew prematurely were requested to return for a final evaluation identical to week 52.

### Efficacy assessments

The outcome variables were changes of total brain and hippocampal volume, regional changes in NAA and MI in relation to baseline at weeks 26 and 52, as well as the global and regional changes of glucose metabolism between baseline and week 52.

### ^18^F–FDG Positron Emission Tomography (PET)

PET scans were acquired on a Siemens-ECAT scanner (Siemens Medical Systems, Erlangen, Germany) 30 min after intravenous injection of 250 MBq of ^18^F-FDG. Imaging was performed in a resting condition with eyes open and ears unoccluded in a dark room with minimal ambient noise. Transmission scans were acquired before the emission scan for attenuation correction. The imaging plane was parallel to the canthomeatal line. The spatial inplane resolution was 4.5 mm and axial resolution was 6 mm full width at half maximum. Using a filtered back-projection method, all images were reconstructed in a 128×128×63 matrix providing a pixel size of 2.5×2.5×2.4 mm.

### MRI

The acquisition of structural scans and CSI was performed in a single session on a 1.5T Philips-Intera scanner (Philips Medical Systems, Best, The Netherlands). The scan protocol included an axial fluid-attenuated inversion recovery (FLAIR) sequence (repetition time (TR) = 6000 ms; echo time (TE) = 130 ms; inversion time (TI) = 1200 ms; field of view (FOV) = 230 mm; matrix = 256×256; slice thickness (THK) = 5 mm), an axial T_2_-weighted fast spin echo (FSE) sequence (TR = 3900 ms; TE = 80 ms; FOV = 230 mm; matrix = 256×256; THK = 5 mm) and a volumetric magnetisation-prepared rapid acquisition gradient echo (MPRAGE) sequence (flip angle = 15°; TR = 20 ms; TE = 4.5 ms; TI = 400 ms) with whole brain coverage. To allow manual segmentation of the hippocampus, the MPRAGE sequence was acquired perpendicularly to the long axis of the hippocampus with a 1.0×1.0 mm inplane resolution and with 1.2 mm-thick partitions. Additionally, for the CSF correction of the CSI data and for the regional analysis of the PET data, a T_1_-weighted true inversion recovery (IR)-FSE sequence (TR = 4400 ms; TE = 15 ms; TI = 350 ms) with a high inplane resolution (0.45×0.90 mm) was performed in an axial orientation.

### Quantitative ^1^H-CSI

CSI was performed in a single axial slice using a point-resolved spectroscopy sequence (PRESS) and a circularly polarised transmit-receive coil. The PRESS sequence was performed with TE = 30 ms, TR = 1500 ms and a 24×24 acquisition matrix. The 15 mm-thick slice had an inplane resolution of 10×10 mm and was positioned to match exactly the five central slices of the IR-FSE sequence. The PRESS sequence was performed slice-selectively instead of selecting a large volume of interest. This enabled full coverage of the parenchyma in the imaging slice but required multiple rest slabs to suppress unwanted fat signal from bone and skull. Water suppression was performed with an adiabatic saturation pulse. Shimming and power optimisation was performed fully automated. To allow for eddy current correction and improved phasing of the spectra, a water-unsuppressed reference scan with a reduced acquisition matrix (12×12) but otherwise identical parameters was performed prior to the water-suppressed scan. To obtain absolute metabolite concentrations, a calibration measurement with a spherical phantom containing 50 mmol/litre NAA and 100 mmol/litre phosphate was performed after each session. To correct for variation of the coil load between patients and the calibration phantom, the coil load for each measurement was determined during the power optimisation process and recorded. The acquisition time for MRI and CSI without calibration was approximately 45 min.

### Analysis of imaging data

#### Glucose metabolism

Regional glucose metabolism was measured manually in predefined regions of interest in the PET images including the frontal, parietal, occipital and temporal lobe, and the basal ganglia. The relative counts obtained from these regions were normalised by the pons activity. Region outlining was performed on the baseline IR-FSE scan, after the baseline and follow-up PET scan had been registered with it. Registration was performed with an affine nine-parameter model following skull stripping of the IR-FSE scan.^24^ The region masks produced at baseline were also used for the analysis of the follow-up PET scans.

#### Brain volumes

Whole brain atrophy and normalised brain volume (NBV) were calculated from the MPRAGE scan using the fully automated structural image evaluation, using normalisation, of atrophy (SIENA) and structural image evaluation, using normalisation, of atrophy (cross sectional) (SIENAX) methods, respectively, which are part of the University of Oxford Functional MRI of the Brain (FMRIB) group’s Software Library (FSL) (http://www.fmrib.ox.ac.uk/fsl).^25^ In addition to its robustness, SIENA provides an error in brain volume change of about 0.2%.^26^ SIENAX determines NBV by extraction of brain tissue, calculation of brain volume and normalisation for subject head size using a volumetric scaling factor. The scaling factor is obtained by an affine registration of the brain image to MNI152 space. The NBV was assessed at baseline only, while the measurement of brain volume change was performed for all subsequent time points. Hippocampal volumes were measured from the T_1_-weighted MPRAGE scans. After image intensity normalisation and registration with the baseline examination, manual tracing of the hippocampal formation was performed on magnified and interpolated coronal sections. Manual tracing was performed blinded to clinical information and time point of examination, in consultation with neuroanatomic atlases.^26^ ^27^ The outlined volume included the hippocampus proper (cornu ammonis CA1 through CA4), gyrus dentatus, subiculum, uncal apex, fimbria and alveus, and excluded the entorhinal cortex (ambient gyrus, parahippocampal gyrus).^7^ ^28^ The hippocampal volume was obtained by averaging the volume of the left and the right hippocampus.

#### Brain metabolites

CSI data were processed voxel by voxel by the fully automated method LCModel.^29^ LCModel analyses an in vivo spectrum as a linear combination of model spectra of metabolite solutions in vitro and provides absolute quantification by reference to an external calibration standard. To account for differences in scanner characteristics between the acquisition of the metabolite solutions in vitro and the in vivo spectra, LCModel uses a calibration factor to scale the in vivo spectra. According to the methodological requirements, the calibration factor was obtained by using LCModel to estimate the known NAA concentration in the calibration phantom and by regarding differences in the coil load factor. Additionally, the low-resolution, unsuppressed CSI data were incorporated in the algorithm to facilitate phasing and eddy-current correction. NAA and MI maps showing the absolute metabolite concentrations were generated from the voxel-wise analysis. These maps were then overlaid on the central slices of the IR-FSE scan, which were virtually aligned with the CSI data. No active registration of the IR-FSE scan and metabolite maps was performed. Regional measurement of metabolite concentration was performed by clustering individual voxels using bespoke in-house tools. Due to the high resolution and excellent T_1_ contrast, the IR-FSE scan also allowed to separate grey matter, white matter and CSF in each spectroscopic voxel to correct for CSF occupancy. The relative CSF occupancy was calculated by fitting three Gaussian functions into the signal intensity distribution function in each voxel.

### Statistical analyses

This study was a first attempt to provide morphological and functional imaging data on the action of memantine in AD in a complex multimodal manner, and was thus designed as a pilot study. All efficacy variables were analysed in a descriptive and exploratory manner. p Values have to be interpreted accordingly, with no adjustments made for multiplicity. The analyses were performed by intention-to-treat. Differences in percentage brain volume change and annual change of normalised glucose metabolism for the whole brain and predefined brain regions between the two groups were assessed using the unpaired Student t test or Mann–Whitney U test, as appropriate. Analysis was performed for changes from baseline to 6 and 12 months. A paired Student t test or the Wilcoxon signed rank test was used for the difference between baseline and final examination overall and within groups. For NAA or MI levels within regions of interest, analysis of covariance including treatment group as the main factor in the model and baseline as covariate was performed. The correlation between glucose metabolism and volumetric measures was determined with the Spearman rank correlation coefficient.

## RESULTS

A total of 37 patients were randomised, of which 36 (1 had claustrophobia in the MRI scanner) received study medication (18 memantine and 18 placebo). Baseline characteristics of the study group are shown in Table 1. The two groups were comparable regarding age, gender, depression score, severity of cognitive symptoms and normalised brain volume.

**Table 1:** Baseline characteristics of patients with Alzheimer disease (AD) in a 52-week, randomised, double-blind, placebo-controlled trial on the effects of memantine on brain morphology and metabolism

Characteristic	Total group (n = 36)	Placebo (n = 18)	Memantine (n = 18)
Age, years	76.2 (5.21)	75.8 (5.70)	76.5 (4.81)
Sex, female	23.0 (63.9)	10.0 (55.6)	13.0 (72.2)
Apolipoprotein-E-ϵ4 allele	16.0 (44.4)	8.0 (44.4)	8.0 (44.4)
Hachinski score	1.1 (1.27)	0.8 (0.99)	1.4 (1.46)
MMSE	19.0 (2.90)	19.3 (2.72)	18.7 (3.12)
GDS	2.4 (0.94)	2.2 (1.00)	2.6 (0.85)
CDR	1.6 (0.56)	1.5 (0.51)	1.6 (0.61)
ADAS-Cog	27.5 (10.59)	27.5 (10.00)	27.6 (11.45)
Normalised brain volume, ml	1298.3 (76.5)	1311.4 (77.5)	1285.9 (75.8)

All data are expressed as mean and standard deviation (SD) except for sex and APO-E-ϵ4 allele, which are expressed as frequency and percentage.

ADAS-Cog, Alzheimer Disease Assessment Scale – cognitive subscale; CDR, Clinical Dementia Rating; GDS, Geriatric Depression Scale; MMSE, Mini-Mental State Examination.

The 26-week follow-up was completed by 32 (89%) patients (16 in each group); 24 (67%) participants (11 on placebo and 13 on memantine) finished the 52-week examination. Overall, 12 (7 placebo and 5 memantine) patients (33.3%) discontinued the study due to adverse events in 4, implantation of pacemaker in 3, technical MRI problems in 2 and non-compliance in 2 subjects. One patient died prior to the 52-week examination. All patients on memantine and 94% of patients on placebo received concomitant medication during the study. In the placebo and memantine group, 33% and 28% received low-dose neuroleptics, respectively; antidepressants were used in 39% and sedatives/hypnotics in 11% of patients in each group. Clinically, the total group was stable until week 26 and declined in all clinical assessments at week 52 (4.2 points on ADAS-Cog, 1.3 on MMSE, 0.4 on CDR and 11 on ADCS-ADL). The 52-week decline in patients on placebo on the ADAS-Cog, MMSE, CDR and ADCS-ADL was 8.2, 2.0, 0.5 and 5.1 points, respectively. With 1.0, 0.7, 0.3 and 5.1 points, the clinical 52-week decline in study participants treated with memantine was slower.

**Figure 1:**
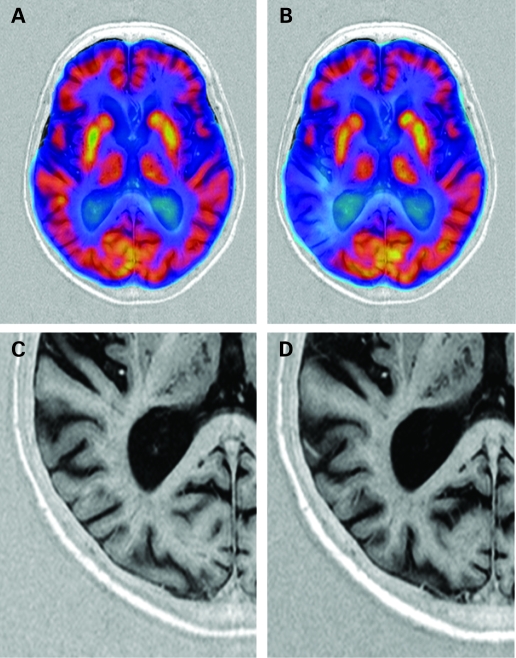
A–D. Baseline (A) and 52-week (B) positron emission tomography (PET) scans registered on MRI in an 85-year-old female study participant who experienced a focal, almost complete loss of glucose utilisation in the right temporoparietal region. Glucose metabolism in other brain regions remained almost unchanged from baseline. The baseline scan demonstrated the typical symmetric temporo-parietal hypometabolism of patients with Alzheimer disease (AD). The coregistered T_1_-weighted MRI scans show little, if any, sulcal enlargement in the area of metabolic loss in this patient between baseline (C) and follow-up (D).

**Figure 2:**
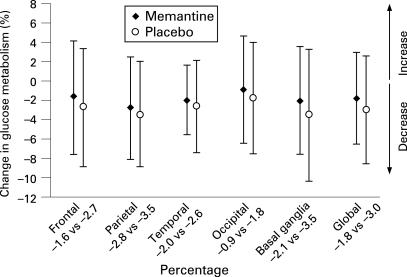
Regional and global changes from baseline in glucose metabolism in patients with Alzheimer disease in a 52-week randomised, double-blind, placebo-controlled trial. Numbers below regions indicate the percentage decline in patients receiving memantine vs those receiving placebo. After 52 weeks, memantine-treated subjects had smaller reductions in all brain regions and globally. Differences did not reach statistical significance in this pilot study.

After 52 weeks, the mean (SD) global decrease in cerebral glucose metabolism from baseline was 2.3% (4.96%); p<0.01. Significant regional metabolic reductions were seen in the parietal (3.1% (5.20%)), basal ganglionic (2.7% (6.00%)) and temporal (2.3% (4.01%)) brain areas (p<0.01 each). Declines in the frontal (2.1% (5.96%)) and occipital (1.3% (5.50%)) areas were non-significant. Longitudinal coregistration of baseline and 52-week follow-up PET studies on MRI reference scans depicted the distribution of functional loss over time. Most AD cases showed a diffuse glucose metabolism decrease involving all brain areas, but some developed substantial asymmetric and focally confined reductions in brain metabolism over time (fig 1 A, B). Memantine-treated subjects showed less annual decline in glucose metabolism in all brain areas compared to placebo (fig 2). The differences were non-significant. In relative terms, the annual decline in global glucose metabolism in patients on memantine was 41% smaller than in those on placebo.

Table 2 shows the changes of MRI volumetric endpoints over time. There was a significant loss of total brain (2.1% per year; p<0.001) and hippocampal (2.7% per year; p<0.01) volumes in all patients. The annual reduction in total brain volume was similar between the groups (2.0% placebo vs 2.3% memantine). At study end, patients on memantine showed 40.6% less hippocampal volume reduction than patients on placebo (2.4% vs 4.0%).

**Table 2:** Changes in MRI volumetric outcome measures from baseline in patients with Alzheimer disease (AD) in a 52-week, randomised, double-blind, placebo-controlled trial of the effects of memantine on brain morphology and metabolism

	Imaging endpoint	Assessment	n	Total group	n	Placebo	n	Memantine
Volumetry	Brain volume change (%)	Week 26	29	−0.6 (1.69)	15	−0.8 (2.04)	14	−0.6 (1.23)
		Week 52	21	−2.1 (2.01)	12	−2.0 (1.92)	9	−2.3 (2.22)
	Hippocampal volume change (%)	Week 26	29	−1.9 (2.82)	15	−2.4 (2.01)	14	−2.0 (2.70)
		Week 52	21	−2.7 (3.64)	12	−4.0 (3.99)	9	−2.4 (2.81)

Numbers are mean (SD). Differences between treatment groups were non-significant based on analysis of covariance (ANCOVA).

There existed no significant relationship between global change in glucose metabolism and percentage change in total brain (r = −0.02) and hippocampal (r = −0.28) volume. Even in the case with almost complete regional drop in glucose metabolism, only minimal sulcal widening was seen at the affected site during the 52-week observational period (fig 1C,D).

CSI was severely affected by patient-induced artefacts, mostly due to patient motion during the 20-min acquisition time. Motion resulted in incomplete fat saturation and marked line-width broadening in most cases. Depending on the brain region, many spectra were excluded from analysis due to unacceptable quality; this decision was made before unblinding. The exclusion rate was lowest in the parietal (5 cases) and highest in the occipital (19 cases) brain area. Analysis of the remaining spectra still yielded a high intra and interindividual variability of all metabolites with no clear trends over time for the entire cohort and patient subgroups (data not shown).

## DISCUSSION

We have demonstrated that multimodal functional and morphological neuroimaging is feasible in a treatment trial of patients with moderate AD. This 52-week study yielded significant reductions in glucose metabolism and total brain and hippocampal volumes in a group of patients with AD with significant clinical deterioration. Our patients on placebo showed similar cognitive decline to that reported in large-scale clinical AD trials,^30^ and their annual 2.0% loss of total brain volume and 4.0% loss of hippocampal volume are also consistent with previous reports,^31^^–^34 which supports the validity of our study.

The greatest problems were posed by CSI. It is important to note here that there was a tremendous difference between single-voxel MRS and CSI regarding motion sensitivity. Obviously, a 20-min acquisition time required per our quantitative CSI protocol was too long for many study participants. In patients who remained still in the scanner, CSI was technically feasible, but data quality was often severely degraded by patient-induced motion artefacts. Typically, head motion resulted in line-width broadening and displacement errors leading to a high rate of spectra of unacceptable quality and to highly variable metabolite concentrations. Thus, low patient compliance can clearly be a major limiting factor when using CSI in longitudinal dementia studies.

Recent developments such as turbo CSI or the incorporation of the SENSE technique reduces acquisition time and may overcome some of the problems we encountered. Until respective studies are available, single-voxel spectroscopy with substantially shorter acquisition times may be preferable in demented patient populations.

We saw no, or at most poor, correlations between metabolic and volumetric cerebral changes over time. Even in the case with almost complete focal loss of metabolic activity in the right temporal-parietal area, sulcul widening was barely present. This suggests long-term latency between functional deterioration and cellular loss in AD, a finding corroborated by studies in presymptomatic familial AD individuals who also showed widespread reductions in glucose metabolism in the relative absence of structural brain changes long before the clinical onset of the disease.^35^

This is also the first investigation utilising neuroimaging techniques to study treatment effects of memantine. Memantine preferentially blocks excessive *N*-methyl-d-aspartic acid (NMDA) receptor activity without disrupting normal receptor activity and is thought to be a neuroprotective agent that attenuates excitotoxicity. Various experiments on cellular and tissue level as well as in animal models support this assumption.^36^ Selection of imaging methods for the current study attempted to cover possible treatment-related effects as comprehensively as possible. Slowing of decline in glucose metabolism and of hippocampal volume loss seen in patients treated with memantine compared to those on placebo is in favour of functional and neuroprotective effects of this substance. However, caution is advised when interpreting these findings, because our study was not powered to detect statistically significant results and we could be dealing with spurious findings. It is important to emphasise that this study was conducted to facilitate planning of larger confirmatory trials. Nevertheless, glucose metabolism in patients treated with memantine was preserved longer in all brain areas, and the effects on PET results and on hippocampal volume were substantial (PET global annual change: 1.8% in patients treated with memantine vs 3% in patients on placebo; hippocampal volume annual percentage change: 2.4% in patients treated with memantine vs 4% in patients on placebo). In relative terms, global cerebral glucose utilisation and hippocampal volume showed a non-significant trend towards a 40% lower annual decline in the memantine than in the placebo group. This result can serve for rough orientation on sample sizes needed. Based on memantine’s 1-year effect on global glucose metabolism and hippocampal volume, the sample sizes required to detect this 40%-reduction in a 1-year trial with a power of 80% (two-sided Student t test at 5% level) are 202 and 70 patients per group, respectively.

The use of multimodal imaging in this study suggests that memantine has functional effects in all brain regions affected by AD while the substance exerted morphological effects only on the hippocampus but not on the whole brain. We certainly cannot exclude that global effects on brain volume may be seen with observation times exceeding 1 year. Hypotheses explaining slower hippocampal degeneration in the absence of effects on total brain volume are difficult to formulate as the mechanisms of neuronal loss in AD are largely unknown. Possibly, the hippocampus may be particularly involved in mild to moderate AD stages studied in the current trial. Another explanation is that memantine exerts more pronounced activity on the hippocampus than on other brain regions because the hippocampus contains a high density of NMDA receptors, particularly in the CA1 area.

In conclusion, the data of this pilot study show that multimodal neuroimaging is feasible in a patient population with mild to moderate AD, and consistent changes over time can be detected by all methods except CSI. Our data suggest that memantine slows the decline in glucose metabolism and slows the progression of hippocampal atrophy supporting its potential disease-modifying effect in AD. Larger trials including PET scanning and hippocampal measurements are warranted to confirm these results.
